# Functional Response, Interference, and Predation Efficiency of *Diomus guilavoguii* (Coleoptera: Coccinellidae) on *Paracoccus marginatus* (Hemiptera: Pseudococcidae)

**DOI:** 10.3390/insects16090971

**Published:** 2025-09-17

**Authors:** Qijing Lin, Guoguo Ruan, Mingjie Tang, Xuanjie Guo, Meixiaoyun Yang, Xingmin Wang, Xiaosheng Chen

**Affiliations:** 1 Department of Forest Protection, College of Forestry and Landscape Architecture, South China Agricultural University, Guangzhou 510642, China; 2 Engineering Research Center of Biological Control, Ministry of Education, Guangzhou 510642, China

**Keywords:** Coccinellidae, ladybird beetle, natural enemy, biological control, functional response

## Abstract

*Paracoccus marginatus* (Hemiptera: Pseudococcidae) poses a significant threat to over 200 plant species, severely impacting agricultural productivity. *Diomus guilavoguii* (Coleoptera: Coccinellidae) is a natural predator of *P. marginatus*. To develop an effective and environmentally friendly management strategy against *P*. *marginatus*, this study investigates the predation relationship between *D. guilavoguii* and *P. marginatus* by focusing on functional response and mutual interference under controlled laboratory conditions (Petri dishes). The results indicated that *D. guilavoguii* exhibits a type II functional response toward *P. marginatus*, with adults of *D. guilavoguii* demonstrating superior efficiency in preying upon *P. marginatus* (the theoretical daily maximum predation rate for female adults of *D. guilavoguii* on young mealybugs is 416.667). However, a decrease in the predators’ search effect was observed with increasing prey density. Additionally, interspecific interference competition intensified as the number of predators increased, resulting in reduced predation efficiency. Consequently, *D. guilavoguii* shows promise biological control agent for the management of *P. marginatus* under laboratory conditions, although further studies in greenhouse and field environments are required to validate its potential in practical pest management.

## Simple Summary

*Paracoccus marginatus* is a polyphagous pest that threatens more than 200 plant species. Currently, the most widely applied biological control methods predominantly utilize parasitoid natural enemies, while the application of predatory natural enemies remains relatively limited. This study investigates the predatory behavior of *Diomus guilavoguii* against *Paracoccus marginatus* by observing functional responses, search effects and interspecific interference effects under controlled laboratory conditions. The results indicate that *Diomus guilavoguii* shows promise as a biological control agent for managing *Paracoccus marginatus* infestations.

## 1. Introduction

The papaya mealybug, *Paracoccus marginatus* Williams & Granara de Willink (Hemiptera: Pseudococcidae), is native to Mexico and Central America [1]. It has rapidly expanded its host range and invaded over 50 countries and regions, adversely affecting agricultural and forestry industries [2]. This species exhibits strong reproductive capacity and remarkable adaptability to diverse environments, making it a significant global invasive pest.

*P. marginatus* mainly congregates on the veins of leaves and fruits, inserting its stylets into plant tissues such as leaf epidermis, fruit skin, or stems to feed on cell sap [3,4,5,6,7]. Meanwhile, it inject toxic substances into plants, causing severe damage including chlorosis, distortion, stunting, premature leaf and fruit drop, honeydew production, sooty mold formation, and potentially plant death [8,9]. Moreover, this insect also acts as a vector of plant viruses such as Piper yellow mottle virus, Cocoa swollen shoot virus, and Papaya ringspot virus [8]. *P. marginatus* infestations can reduce crop yields by 10–60%, and in extreme cases up to 90% [10]. Therefore, it is imperative to implement suitable pest control measures to prevent further damage by *P. marginatus*.

Chemical control was once regarded as an effective method for preventing plant infestation by *P. marginatus*. Early pesticides commonly used to control *P. marginatus* included methyl demeton, quinalphos, fenthion, acephate, dimethoate, malathion, and white mineral oils [11]. However, due to the wax coating and cottony ovisac of the mealybug, it is challenging to develop effective strategies to ensure adequate chemical penetration [7,11]. Some newer insecticides, such as buprofezin, imidacloprid, thiomethoxam, spirodiclofen, pyridaben, chlorfenapyr, and plant essential oils (neem, citrus, garlic, and castor), have shown promise in controlling the papaya mealybug [11,12,13,14]. Nevertheless, chemical pesticides often have residual effects and can cause environmental pollution [7,11,12,14]. Additionally, frequent use of chemical pesticides can lead to pest resistance and may harm non-targeted insects and natural enemies, complicating pest management efforts [15].

Compared with chemical control, biological control aligns more closely with the principles of sustainable development and environmental protection. Commonly employed biological control agents for managing papaya mealybug include parasitoids such as *Acerophagus papayae* Noyes & Schauff, *Anagyrus loecki* Noyes & Menezes, and *Pseudleptomastix mexicana* Noyes & Schauff (Hymenoptera: Encyrtidae) [15,16,17,18,19,20]. These parasitoids have been shown to significantly reduce papaya mealybug populations in the field, thereby mitigating potential economic losses [16,18,19]. However, there are instances where the parasitoid populations fail to recover adequately after release [16,17].

As an important natural enemy of insects, predatory ladybird beetles have been utilized in biological control for nearly 120 years. Due to their broad prey spectrum and adaptability to new environments, they play a crucial role in the management of pest populations [21]. Xia et al. [22] demonstrated that *Chilocorus kuwanae* (Coleoptera: Coccinellidae), as a natural predator of various pests, can prey on 27 species across 5 families. Li et al. [23] reported that *Coccinella septempunctata*, *Chilocorus kuwanae*, *Harmonia axyridis*, *Coccinula quatuordecimpustulata*, *Rodolia limbata* and *Synharmonia bissexnotata* (Coleoptera: Coccinellidae) all exhibit biocontrol potential against *Matsucoccus sinensis* (Hemiptera: Margarodidae). Similarly, the feeding behavior of *Cryptolaemus montrouzieri* (Coleoptera: Coccinellidae) has been well studied, showing its strong efficiency in suppressing mealybug populations, which provides a valuable reference for evaluating other coccinellid predators [24,25]. Information regarding the host range of species within the genus *Diomus* remains limited; however, existing data suggest a clear preference for mealybugs [26]. *Diomus guilavoguii* Duverger, native to Conakry, Guinea [27], was first recorded in Guangzhou, China in 2022, where it was observed preying extensively on *P. marginatus* [28]. However, detailed studies on the predatory efficacy of *D. guilavoguii* against *P. marginatus* are unavailable to date. Therefore, this study aims to evaluate the predation rate, functional response, search effect, and mutual interference of *D. guilavoguii* when preying on *P. marginatus*.

## 2. Material and Methods

### 2.1. Paracoccus marginatus

*P. marginatus* was collected from infested *Jatropha integerrima* (Malpighiales: Euphorbiaceae) leaves on the campus of South China Agricultural University (SCAU). Subsequently, *J. integerrima* seedlings were inoculated with *P. marginatus* to propagate the pest for further study.

### 2.2. Diomus guilavoguii

*D*. *guilavoguii* was collected from *J. integerrima* leaves infested by *P. marginatus* on the campus of South China Agricultural University (SCAU)*. D. guilavoguii* were kept in a square cage (60 cm × 60 cm × 60 cm) and fed with *P. marginatus* on fresh *J. integerrima* leaves. After oviposition, the eggs on the leaves were transferred to disposable polystyrene Petri dishes (3.5 cm in diameter, 1 cm in height; Guangzhou Qianhui Reagent Instrument Co., Ltd., Guangzhou, China) containing moderately sized *P. marginatus* leaves and moistened cotton wool. The Petri dishes were wrapped with cling film to prevent desiccation and larval escape. The leaves with prey were changed daily to maintain cleanliness and ensure proper feeding of the predators. Larvae were observed regularly until they emerged as adults. Adults were then transferred using a fine brush into new cages for mating and subsequent oviposition.

### 2.3. Functional Response

Third-instar and fourth-instar larvae, male and female adults of *D. guilavoguii* were selected and kept without food for 24 h in separate disposable polystyrene Petri dishes. The selected individuals included larvae within two hours after molting and adults within 24 to 48 h after eclosion. Subsequently, all individuals were exposed to varying densities of 3rd- to 4th-instars nymphs and adults (hereafter referred to as “late-instar nymphs and adults”) of *P. marginatus* (1, 3, 5, 7, 9, 11, 13, 15, 17 and 19). Third- and fourth-instar larvae were also exposed to different densities of 1st- to 2nd-instar nymphs (hereafter referred to as “young nymphs”) of *P. marginatus* (10, 20, 30, 40, 50, 60, 70, 80, 90 and 100). Male and female adults of *D. guilavoguii* were also exposed to different densities of young nymphs of *P. marginatus* (80, 120, 160, 200, 240, 280, 320, 360, 400 and 440). Leaves (approximately 6 cm in length after being trimmed into square-shaped sections) of *J. integerrima* were placed in disposable polystyrene Petri dishes (9 cm in diameter; Guangzhou Qianhui Reagent Instrument Co., Ltd., Guangzhou, China), and predators were transferred to these dishes using a fine brush. Each dish contained one predator, a damp cotton ball, and was covered with plastic wrap pierced with needles. All dishes were placed in an artificial climate chamber at a temperature of 26 ± 1 °C with a 14 h photophase. After 24 h of exposure, the remaining number of *P. marginatus* was counted. Each treatment was repeated five times.

### 2.4. Mutual Interference

The predators, which had been starved for 24 h, were divided into five groups, and placed in separate disposable polystyrene Petri dishes (9 cm in diameter), each containing fresh *J. integerrima* leaves. Each dish contained 1, 2, 3, 4, or 5 predators, respectively. The 3rd and 4th instar larvae of *D. guilavoguii* were exposed to either 200 young nymphs of *P. marginatus* or 30 late-instar nymphs and adults of *P. marginatus*, while adult females and males were exposed to either 500 young nymphs of *P. marginatus* or 30 late-instar nymphs and adults of *P. marginatus*. Each dish had a moist cotton plug and was covered with plastic wrap punctured with needles to allow ventilation. All dishes were placed in an artificial climate chamber (RXZ-500A, Ningbo Jiangnan Instrument Factory, Ningbo, China) maintained at 26 ± 1 °C with a 14 h photoperiod. After 24 h of exposure, the remaining number of *P. marginatus* was recorded. Each treatment was repeated five times.

### 2.5. Analysis of Data

Data on predation rate and the amount of *D. guilavoguii* feeding on *P. marginatus* were analyzed using one-way analysis of variance (ANOVA) to test for differences among treatments. When significant differences were detected (*p* < 0.05), means were compared using Tukey’s Honestly Significant Difference (HSD) test. Statistical analyses were conducted using SPSS Statistics 25.0 software (IBM Corp., Armonk, NY, USA).

The type of functional response was determined by nonlinear logistic regression using the “frair” package within the “R” software version 4.0.2 (R Foundation for Statistical Computing, Vienna, Austria) [29]. The polynomial function that describes the relationship between $N_{a}/N_{0}$ and $N_{0}$ was derived using the following equation:

$$\frac{N_{a}}{N_{0}}=\frac{exp(P_{0}+P_{1}N_{0}+P_{2}N_{0}^{2}+P_{3}N_{0}^{3})}{1+exp(P_{0}+P_{1}N_{0}+P_{2}N_{0}^{2}+P_{3}N_{0}^{3})}$$

In this equation, $N_{a}$ presents the number of prey consumed, $N_{0}$ denotes the initial number of prey provided, and *P*_0_, *P*_1_, *P*_2_, and *P*_3_, are the constant, linear, quadratic, and cubic coefficients (maximum likelihood estimates), respectively, related to the slope of the curve. A positive $P_{1}$ and a negative $P_{2}$ in polynomial logistic regression indicate a type III functional response. Conversely, when $P_{1}$ is negative, the number of prey consumed initially decreases with increasing prey availability, which characterizes a type II functional response [30].

The selection of the functional response model entails the application of polynomial logistic regression. For a clear type II response, both the disk equation [31] and the random attack equation [32] can be used to estimate handling time (*T_h_*) and attack rate (*a*). Given that the disk equation requires constant prey density during experiments, we opted for the random attack equation as our model.

$$N_{a}=N_{0}\left\{1-exp\left[a\left(T_{h}N_{a}-T\right)\right]\right\}$$

Here, $N_{a}$ is the number of prey consumed, $N_{0}$ is the initial number of prey provided, *a* represents the attack rate, $T_{h}$ refers to the time required for capturing, subduing, and consuming prey (digestion may continue after ingestion but does not necessarily prevent subsequent predation) [33,34], T indicates the total exposure time of the predator to prey, typically set at 24 h. The attack rate (*a*) and handling time ($T_{h}$) were estimated using nonlinear regression, following the method of Rogers [32], and compared using the “frair_compare” function from the “frair” package in “R” software [29]. Additionally, the maximum predation rate $T/T_{h}$ was also calculated.

The search effect (*S*) is related to the prey density (*N*), and is defined as the proportion of the available prey population that a predator effectively encounters and attacks per unit time [35,36]. It can be expressed by the formula $S=a/(1+aT_{h}N)$, where S represents the searching effect, while the parameters *a* and $T_{h}$ are derived from the type II functional response.

The predation efficiency (*E*) is affected by the predator’s own density (*P*). As the predator density increases, the interference effect also intensifies. The formula for predation efficiency is $\;E=N_{a}/NP$, where *N_a_* represents the number of prey captured [37]. The mutual interference equation is $E=QP^{-m}$, where *Q* denotes the search constant and *m* presents the interference coefficient. The apportionment competition intensity (*I*) fits the formula $I=\left(E_{1}-E_{p}\right)/E_{1}$, where $E_{1}$ is the predation efficiency of a single predator, and $E_{p}$ is the predation efficiency of a group of *p* predator. The mutual interference coefficient (*m*) among predators was measured using the Hassell and Varley [38] model: logE=logQ−mlogP. This was achieved by fitting the data to the equation using a linear regression procedure in IBM SPSS Statistics 25.

## 3. Results

### 3.1. Predation Rate of Diomus guilavoguii Feeding on Paracoccus marginatus

Through analysis of variance, there was a statistically significant difference in predation rates observed for *D. guilavoguii* at different densities of *P. marginatus*. The results showed that the overall consumption percentage of mealybug by *D. guilavoguii* decreased as prey density increased. Specifically, at the highest prey density tested in the experiment, the predation rate for adult *D. guilavoguii* was more than 0.22, while the predation rate for larvae was less than 0.28 (Table 1 and Table 2).

### 3.2. Functional Response of D. guilavoguii Feeding on P. marginatus

In these experiments, *D. guilavoguii* showed predatory capability against both nymph and adult stages of *P. marginatus* (Table 3). The results of the logistic regression analysis were significant (*p* < 0.05), with a negative linear coefficient ($P_{1}$), indicating that *D. guilavoguii* displays a type II functional response.

**Figure 1:**
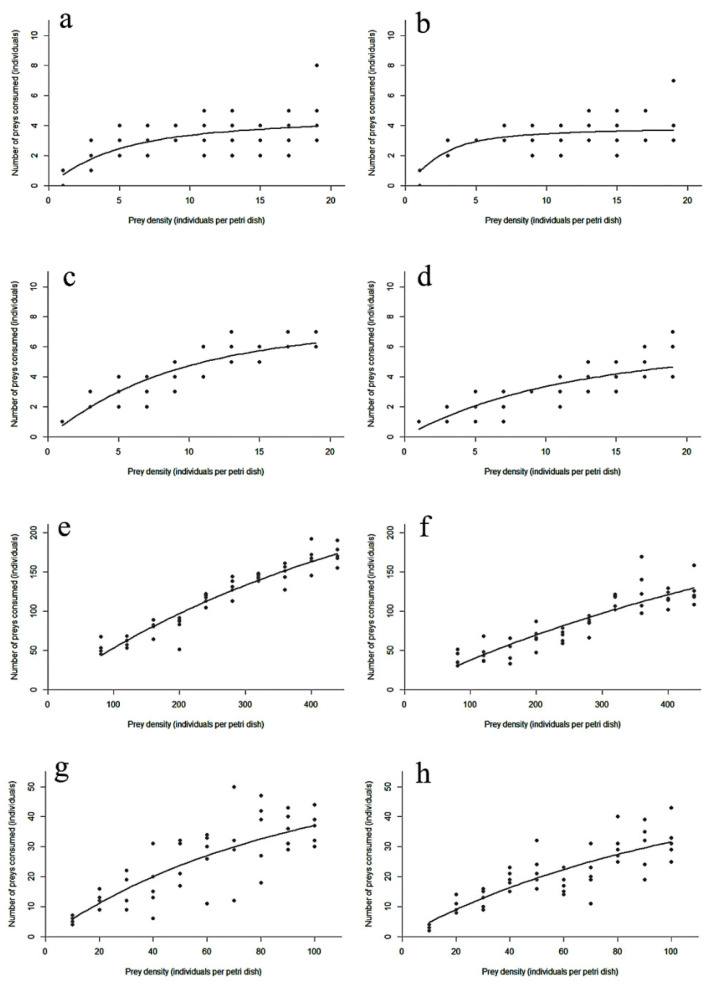
*Paracoccus marginatus* consumption by *Diomus guilavoguii* at different prey densities. Note: (**a**) female adults of *Diomus guilavoguii* on late-instar nymphs and adults of *Paracoccus marginatus*, (**b**) male adults of *Diomus guilavoguii* on late-instar nymphs and adults of *Paracoccus marginatus*, (**c**) 4th larvae of *Diomus guilavoguii* on late-instar nymphs and adults of *Paracoccus marginatus*, (**d**) 3rd larvae of *Diomus guilavoguii* on late-instar nymphs and adults of *Paracoccus marginatus*, (**e**) female adults of *Diomus guilavoguii* on young nymphs of *Paracoccus marginatus*, (**f**) male adults of *Diomus guilavoguii* on young nymphs of *Paracoccus marginatus*, (**g**) 4th larvae of *Diomus guilavoguii* on young nymphs of *Paracoccus marginatus*, (**h**) 3rd larvae of *Diomus guilavoguii* on young nymphs of *Paracoccus marginatus*.

The functional response curves for larvae and adults of *D. guilavoguii* feeding on *P. marginatus* at different ages and densities are shown in Figure 1. Initially, the consumption rate increased sharply with prey density before eventually plateauing.

The instantaneous attack rate (*a*) of *D. guilavoguii* preying upon young nymphs of *P. marginatus* did not differ significantly between developmental stages, except for male adults, which exhibited a significantly lower (*a*) value. However, when preying on late-instar nymphs and adult mealybugs, the attack rate showed statistically different (*a*) values, with female adults (1.4124), male adults (3.4485), 4th instar larvae (1.3898), and 3rd instar larvae (0.7280).

At the same prey age, the predation rate of 4th instar larvae was significantly higher than that of 3rd instar larvae, and female adults also had a significantly higher predation rate than male adults. As the age of the mealybug increased, the handling time of *D. guilavoguii* decreased. For 1st to 2nd instar mealybugs, female adults spent the shortest time capturing, ingesting each prey item compared to other stages, while larvae required more time. For 3rd to 4th instar mealybugs, 4th instar larvae of *D. guilavoguii* performed better in terms of the time spent capturing, ingesting each prey item compared to adults and 3rd instar larvae. The maximum predation rate ($T/T_{h}$) to 1st to 2nd instar mealybugs was significantly higher in adults (female: 416.67, male: 400) compared to larval stages (3rd instar: 64.52, 4th instar: 70.93). Conversely, for 3rd to 4th instar mealybugs, the maximum predation rate of adults (female: 4.699, male: 3.925) was significantly lower than that of larval stages (3rd instar: 8.772, 4th instar: 7.576) (Table 4).

### 3.3. Search Effects of D. guilavoguii Feeding on P. marginatus

**Figure 2:**
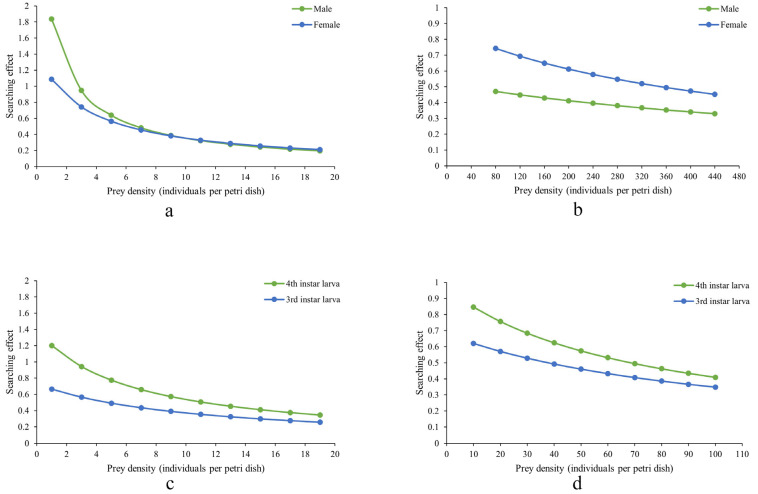
Search effects of *Diomus guilavoguii* feeding on *Paracoccus marginatus.* Note: (**a**) adults of *Diomus guilavoguii* on late-instar nymphs and adults of *Paracoccus marginatus*, (**b**) adults of *Diomus guilavoguii* on young nymphs of *Paracoccus marginatus*, (**c**) larvaes of *Diomus guilavoguii* on late-instar nymphs and adults of *Paracoccus marginatus*, (**d**) larvae of *Diomus guilavoguii* on young nymphs of *Paracoccus marginatus*.

Figure 2 shows that the search effects of *D. guilavoguii* feeding on *P. marginatus* decreased with increasing prey density. In addition, the reduction in search effects was more pronounced for late-instar nymphs and adults of *P. marginatus* compared to younger nymphs, suggesting that higher prey density had a more significant impact on the predatory behavior of ladybirds targeting older stages of the prey. In the case of young nymphs of *P. marginatus*, female adults of *D. guilavoguii* consistently exhibit a higher search effect than male adults. Meanwhile, at the same instar stage and prey density, fourth instar larvae of *D. guilavoguii* demonstrate a higher search effect compared to third instar larvae. These differences can be attributed to variations in handling time, as shorter handling times in female adults and fourth-instar larvae contribute to higher searching efficiency. Since search effect values are derived from the type II functional response model (based on attack rate (*a*) and handling time $T_{h}$), statistical differences among developmental stages are reflected in the comparisons of these parameters. Female adults and fourth-instar larvae exhibited significantly higher attack rates than third-instar larvae (*p* < 0.05), which accounts for their consistently higher search effects across all prey densities.

### 3.4. Mutual Interference of D. guilavoguii Feeding on P. marginatus

From the perspective of prey consumed per predator, when there is only one predator, the predation rate per predator reached its peak. When five predators are present in a group, the predation rate per predator drops to its lowest point. As predator density increases, both the daily predation and average predation efficiency of *D. guilavoguii* against *P. marginatus* decrease, while competition intensifies. At the same predator density, the average predation efficiency of *D. guilavoguii* against elder *P. marginatus* is lower than that against younger nymphs, and the competition intensity from elder mealybug is also lower than that from younger nymphs.

As shown in Table 5 and Table 6, the maximum daily predation of female adult *D. guilavoguii* on older nymphs was 7.6 per day ($E=0.2523P^{-0.802}$), while on younger nymphs it was 132.6 per day ($E=0.2495P^{-0.528}$). For male adults, the maximum daily predation on older nymphs was 5.25 per day ($E=0.1879P^{-0.724}$), and on younger nymphs it was 92.6 per day ($E=0.1799P^{-0.446}$). The 4th instar larvae had a maximum daily predation of 5 on older nymphs ($E=0.1750P^{-0.448}$) and 47 on younger nymphs ($E=0.2399P^{-0.29}$). Third instar larvae showed a maximum daily predation of 3.8 on older nymphs ($E=0.1247P^{-0.484}$) and 40.6 on younger nymphs ($E=0.2037P^{-0.263}$).

The mutual interference parameter (*m*) for female adults preying on late-instar nymphs and adults of mealybugs was the highest, while that for 3rd instar larvae preying on 1st to 2nd instar mealybugs was the lowest.

## 4. Discussion

Functional response has been widely used to evaluate the predation ability of natural enemies. However, no studies have yet investigated the functional response of *D. guilavoguii* toward *P. marginatus*. Our study revealed that both larvae and adults of *D. guilavoguii* exhibit a type II functional response when feeding on *P. marginatus* under laboratory conditions, a pattern also observed in *Harmonia axyridis* preying on various prey species [39,40].

In a type II functional response, the attack rate and handling time are critical determinants of predation efficiency. The attack rate of fourth-instar larvae and female adults is higher than that of other developmental stages of *D. guilavoguii*, indicating a stronger predation capacity during these stages. This pattern is consistent with the feeding behavior of *Cryptolaemus montrouzieri* when preying on *P. marginatus* [25]. The increased predation ability in these stages may be attributed to the higher nutritional demands of fourth-instar larvae preparing for pupation and female adults undergoing reproductive development. The theoretical maximum daily predation rate, which is inversely related to handling time, further reflects the predation efficiency of *D. guilavoguii*. Notably, adults consumed significantly more young nymphs than larvae, demonstrating a higher predatory capability in the adult stage. This observation corresponds to the predation behavior of *Xylocoris sordidus* (Hemiptera: Anthocoridae) feeding on *Enneothrips enigmaticus* (Thysanoptera: Thripidae) [41]. Moreover, *D. guilavoguii* exhibited a preference for younger nymphs over older mealybugs, a trend also reported in *Arma chinensis* (Hemiptera: Pentatomidae) preying on *Henosepilachna vigintioctopunctata* (Coleoptera: Coccinellidae) [42]. Additionally, as prey density increases, the predation rate per individual decreases and the total number of prey consumed reaches a plateau, a phenomenon similarly observed in *Mallada basalis* (Neuroptera: Chrysopidae) feeding on *Spodoptera frugiperda* (Lepidoptera: Noctuidae) [43]. These findings suggest that for optimal biological control using *D. guilavoguii*, they should be released early during a *P. marginatus* outbreak.

Moreover, female adults of *D. guilavoguii* consistently exhibit a higher search effect than males when preying on young nymphs of *P. marginatus*. Additionally, 4th-instar larvae show greater search effect than 3rd-instar larvae on the same prey stage, a pattern also observed in *Harmonia axyridis* preying on *Rhopalosiphum nymphaeae* (Hemiptera: Aphididae) [44]. A higher searching effect suggests that natural enemies can regulate prey populations even at low densities, highlighting their potential effectiveness in controlling pest outbreaks during the early stages of population growth. In other words, female adults are more effective than males in preying on young mealybugs, and under the same prey stage and density, 4th-instar larvae exhibit stronger predatory capacity compared to 3rd-instar larvae.

The mutual interference experiments revealed that increased predator density intensifies intraspecific competition, leading to a reduced per capita predation rate. Comparable effects have been documented in other predator-prey systems, such as *Orius tantillus* (Hemiptera: Anthocoridae) preying on *Thrips hawaiiensis* (Thysanoptera: Thripidae) [45]. Regarding young nymphs of *P. marginatus*, when the density of *D. guilavoguii* reached the maximum level (5 individuals per Petri dish), the intensity of intraspecific competition per ladybird was highest among female adults, which may be attributed to variations in predation capacity across developmental stages.

According to Hassell and Varley, mutual interference can play an important role in regulating host population dynamics [38]. In this context, the observed interference in *D. guilavoguii* suggests that although increased predator density can suppress *P. marginatus* populations, excessive intraspecific competition may diminish individual predation efficiency, thereby preventing uncontrolled host depletion. This dynamic indicates a potential mechanism through which predator interference may contribute to long-term pest population regulation. While our results indicate that increased predator density reduces the individual efficiency of *D*. *guilavoguii* due to mutual interference, predator density can be effectively managed in practical orchard settings. In practice, augmentative releases of coccinellid predators are a widely adopted strategy in integrated pest management programs, offering a means to optimize predator numbers, minimize interference, and ensure effective pest suppression [46,47].

However, this study was conducted under controlled indoor conditions using Petri dishes with fixed prey density, which may artificially enhance predation efficiency. In natural environments, preys are typically dispersed across various parts of host plants, thereby increasing the time and effort required for predators to locate them [36]. Consequently, the actual predation rate in the field is likely lower than that observed in laboratory conditions, which often deviates from the results of functional response studies conducted in controlled chambers [48]. Moreover, the predation capacity of natural enemies in the field is also affected by multiple factors. For example, higher pest densities can reduce predator dispersal and intensify both intra- and interspecific competition, thereby lowering individual predation efficiency [49]. Additionally, environmental factors such as weather conditions, temperature, host plants and the presence of multiple prey species also play significant roles [50]. Therefore, future studies should take into account various natural factors, such as abiotic conditions (including temperature, humidity, and photoperiod), prey density, prey species diversity, and interspecific competition among predators, in order to gain a more comprehensive understanding of the interactions that affect predation dynamics. In this regard, we intend to investigate higher prey densities under greenhouse or semi-field conditions to confirm whether the asymptotic predation rate observed in these settings differs from that estimated in laboratory environments. Greenhouse or semi-field experiments involving potted plants or field cages can effectively simulate natural prey distribution patterns and plant structures, thereby facilitating a more accurate assessment of predator searching efficiency and handling time. The integration of these experimental approaches with field release trials will be essential for verifying whether the parameters derived from laboratory studies can be successfully applied to achieve practical pest suppression. Ultimately, combining laboratory-based models with greenhouse and field evaluations will establish a more robust basis for incorporating *D*. *guilavoguii* into integrated pest management (IPM) programs.

In conclusion, this study demonstrates the potential of that *D. guilavoguii* as a biological control agent against *P. marginatus* under laboratory conditions. However, further investigation is required to evaluate its efficacy in practical field applications.

## Figures and Tables

**Table 1:** Predation rate and amount of *Diomus guilavoguii* feeding on young nymphs of *Paracoccus marginatus*.

Female Adult of *D*. *guilavoguii*			Male Adult of *D*. *guilavoguii*			4th Instar Larvae of *D*. *guilavoguii*			3rd Instar Larvae of *D*. *guilavoguii*		
Prey Density	Predation	Predation Rate	Prey Density	Predation	Predation Rate	Prey Density	Predation	Predation Rate	Prey Density	Predation	Predation Rate
80	53.4 ± 3.709 f	0.67 ± 0.046 a	80	39.2 ± 3.967 d	0.49 ± 0.05 a	10	5.80 ± 0.583 c	0.58 ± 0.058 a	10	3.20 ± 0.374 f	0.32 ± 0.037 ab
120	60.6 ± 2.581 ef	0.51 ± 0.022 b	120	46.4 ± 5.819 d	0.39 ± 0.048 ab	20	11.80 ± 1.319 cd	0.59 ± 0.066 a	20	10.00 ± 1.140 ef	0.50 ± 0.057 a
160	75.8 ± 5.044 ef	0.47 ± 0.032 b	160	49.6 ± 5.758 d	0.31 ± 0.036 b	30	16.20 ± 2.437 cd	0.54 ± 0.081 a	30	12.6 ± 1.364 def	0.42 ± 0.045 ab
200	80.2 ± 7.419 e	0.4 ± 0.037 b	200	67 ± 6.427 cd	0.34 ± 0.032 b	40	17.00 ± 4.159 bcd	0.43 ± 0.104 a	40	19.2 ± 1.356 cde	0.48 ± 0.034 ab
240	115.2 ± 3.184 d	0.48 ± 0.013 b	240	68.4 ± 3.501 cd	0.29 ± 0.015 b	50	26.60 ± 3.172 abc	0.53 ± 0.063 a	50	22.4 ± 2.731 abcd	0.45 ± 0.055 ab
280	130.6 ± 5.278 cd	0.47 ± 0.019 b	280	84 ± 4.764 bc	0.3 ± 0.017 b	60	26.80 ± 4.188 abc	0.45 ± 0.07 a	60	17.6 ± 1.6 de	0.29 ± 0.027 b
320	143.2 ± 1.772 c	0.45 ± 0.006 b	320	113.4 ± 3.919 ab	0.35 ± 0.012 ab	70	27.00 ± 7.099 abc	0.39 ± 0.101 a	70	20.8 ± 3.231 bcde	0.3 ± 0.046 b
360	147.6 ± 5.946 ab	0.41 ± 0.017 b	360	127 ± 12.763 a	0.35 ± 0.035 ab	80	34.60 ± 5.297 ab	0.43 ± 0.066 a	80	30.4 ± 2.6 abc	0.38 ± 0.033 ab
400	168 ± 7.543 a	0.42 ± 0.019 b	400	117 ± 4.626 a	0.29 ± 0.012 b	90	35.80 ± 2.634 a	0.40 ± 0.029 a	90	29.80 ± 3.652 ab	0.33 ± 0.041 ab
440	172 ± 5.822 a	0.39 ± 0.013 b	440	126 ± 8.509 a	0.29 ± 0.019 b	100	36.40 ± 2.502 a	0.36 ± 0.025 a	100	32.20 ± 3.007 a	0.32 ± 0.300 ab

Note: Different lowercase letters following the data within the same column denote significant differences among different prey densities by HSD Test (*p* < 0.05).

**Table 2:** Predation rate and amount of *Diomus guilavoguii* feeding on late-instar nymphs and adults of *Paracoccus marginatus*.

Female Adult of *D*. *guilavoguii*			Male Adult of *D*. *guilavoguii*			4th Instar Larvae of *D*. *guilavoguii*			3rd Instar Larvae of *D*. *guilavoguii*		
Prey Density	Predation	Predation Rate	Prey Density	Predation	Predation Rate	Prey Density	Predation	Predation Rate	Prey Density	Predation	Predation Rate
1	0.60 ± 0.245 c	0.60 ± 0.245 a	1	0.80 ± 0.200 b	0.80 ± 0.200 ab	1	1.00 ± 0.000 e	1.00 ± 0.000 a	1	1.00 ± 0.000 g	1.00 ± 0.000 a
3	1.60 ± 0.400 bc	0.53 ± 0.133 a	3	2.60 ± 0.245 b	0.87 ± 0.0802 a	3	2.20 ± 0.200 de	0.73 ± 0.067 b	3	1.40 ± 0.245 fg	0.47 ± 0.082 b
5	3.00 ± 0.316 ab	0.60 ± 0.063 a	5	3.00 ± 0.000 b	0.60 ± 0.000 abc	5	3.80 ± 0.374 c	0.76 ± 0.0375 b	5	1.80 ± 0.374 efg	0.36 ± 0.075 b
7	3.00 ± 0.316 ab	0.43 ± 0.045 a	7	3.40 ± 0.245 a	0.49 ± 0.035 bcd	7	3.40 ± 0.245 cd	0.49 ± 0.035 c	7	2.20 ± 0.374 defg	0.31 ± 0.053 b
9	3.40 ± 0.245 ab	0.38 ± 0.027 a	9	3.20 ± 0.374 a	0.36 ± 0.042 cd	9	4.20 ± 0.663 c	0.47 ± 0.074 c	9	3.00 ± 0.000 cdef	0.33 ± 0.00 b
11	3.20 ± 0.583 ab	0.29 ± 0.053 a	11	3.00 ± 0.316 a	0.27 ± 0.029 cd	11	4.40 ± 0.400 bc	0.4 ± 0.036 c	11	3.20 ± 0.374 bcde	0.29 ± 0.034 b
13	3.40 ± 0.510 ab	0.26 ± 0.039 a	13	3.60 ± 0.400 a	0.28 ± 0.031 cd	13	6.20 ± 0.374 a	0.48 ± 0.029 c	13	3.60 ± 0.400 bcd	0.28 ± 0.031 b
15	3.20 ± 0.374 ab	0.21 ± 0.025 a	15	3.40 ± 0.510 a	0.23 ± 0.034 d	15	5.80 ± 0.200 ab	0.39 ± 0.013 c	15	4.00 ± 0.316 abc	0.27 ± 0.021 b
17	3.40 ± 0.510 ab	0.20 ± 0.030 a	17	3.40 ± 0.400 a	0.20 ± 0.024 d	17	6.40 ± 0.245 a	0.38 ± 0.014 c	17	4.80 ± 0.374 ab	0.28 ± 0.022 b
19	4.80 ± 0.860 a	0.25 ± 0.045 a	19	4.20 ± 0.735 a	0.22 ± 0.039 d	19	6.40 ± 0.245 a	0.34 ± 0.013 c	19	5.40 ± 0.600 a	0.28 ± 0.032 b

Note: Different lowercase letters following the data within the same column denote significant differences among different prey densities by HSD Test (*p* < 0.05).

**Table 3:** Maximum likelihood estimates for logistic regression of functional response of *Diomus guilavoguii* on *Paracoccus marginatus*.

Predator	Prey	Maximum Likelihood Estimate (±SE)	*p*-Value	Z-Value	Functional Response Type
Female adult of *D*. *guilavoguii*	3 to 4 instar	−0.0970 ± 0.0210	<0.001	−4.6194	Type II
	1 to 2 instar	−0.0016 ± 0.0002	<0.001	−9.028	Type II
Male adult of *D*. *guilavoguii*	3 to 4 instar	−0.1337 ± 0.0216	<0.001	−6.1933	Type II
	1 to 2 instar	−0.0010 ± 0.0002	<0.001	−5.6392	Type II
4th instar larvae of *D*. *guilavoguii*	3 to 4 instar	−0.0707 ± 0.0196	<0.001	−3.6091	Type II
	1 to 2 instar	−0.0098 ± 0.0024	<0.001	−4.0538	Type II
3rd instar larvae of *D*. *guilavoguii*	3 to 4 instar	−0.0453 ± 0.0204	<0.05	−2.2146	Type II
	1 to 2 instar	−0.0083 ± 0.0025	<0.001	−3.292	Type II

**Table 4:** Predatory functional responses of *Diomus guilavoguii* feeding on *Paracoccus marginatus*.

Predator	Prey	Instantaneous Attack Rate *a*		Handling Time *T_h_* (*h*)		Theoretical Daily Maximum Predation *T/T_h_*
		Maximum Likelihood Estimate (±SE)	95% Confidence Interval	Maximum Likelihood Estimate (±SE)	95% Confidence Interval	
Female adult	Late-instar nymphs and adults	1.4124 ± 0.4794 ab	0.809~2.826	0.2128 ± 0.0338 ab	0.135~0.264	4.699
	Young nymphs	0.8655 ± 0.0411 a	0.724~1.070	0.0024 ± 0.0002 a	0.002~0.003	416.667
Male adult	Late-instar nymphs and adults	3.4485 ± 1.3382 a	11.549~20.670	0.2548 ± 0.0257 a	0.203~0.295	3.925
	Young nymphs	0.5181 ± 0.0285 b	0.417~0.693	0.0025 ± 0.0004 a	0.001~0.004	400.000
4th instar larvae	Late-instar nymphs and adults	1.3898 ± 0.3920 ac	1.023~2.047	0.1140 ± 0.0247 c	0.090~0141	8.772
	Young nymphs	0.9610 ± 0.0955 a	0.693~1.328	0.0140 ± 0.0020 b	0.008~0.021	71.428
3rd instar larvae	Late-instar nymphs and adults	0.7280 ± 0.2442 bc	0.450~1.457	0.1320 ± 0.0481 bc	0.053~0.209	7.576
	Young nymphs	0.6792 ± 0.0726 a	0.545~0.922	0.0140 ± 0.0029 b	0.006~0.022	71.428

Note: Different lowercase letters following the data denote significant differences in the instantaneous attack rate or the handling time between different stages of *D. guilavoguii* to the same developmental stage of *P. marginatus* by Z-test (*p* < 0.05).

**Table 5:** Mutual interference of *Diomus guilavoguii* feeding on late-instar nymphs and adults of *Paracoccus marginatus*.

Predator	Intraspecific Interference Equation	Search Constant (*Q*)	Interference Coefficient (*m*)	Predator Density	Prey Density	Prey Killed by per Predator	Predation Rate	Intensity of Scramble Competition (*I*)
Female adult	*E* = 0.2523*P*^−0.802^	0.2523	0.802	1	30	7.60	0.2533	0.0000
				2	30	4.40	0.1467	0.4211
				3	30	3.20	0.1067	0.5789
				4	30	2.55	0.0850	0.6645
				5	30	2.04	0.0680	0.7315
Male adult	*E* = 0.1879*P*^−0.724^	0.1879	0.724	1	30	5.25	0.1750	0.0000
				2	30	3.38	0.1125	0.3571
				3	30	2.50	0.0833	0.5238
				4	30	2.00	0.0667	0.6190
				5	30	1.75	0.0583	0.6667
4th instar larvae	*E* = 0.1750*P*^−0.488^	0.1750	0.488	1	30	5.00	0.1667	0.0000
				2	30	4.10	0.1367	0.1800
				3	30	3.20	0.1067	0.3600
				4	30	2.75	0.0917	0.4500
				5	30	2.20	0.0733	0.5600
3rd instar larvae	*E* = 0.1247*P*^−0.484^	0.1247	0.484	1	30	3.80	0.1267	0.0000
				2	30	2.80	0.0933	0.2632
				3	30	2.13	0.0711	0.4386
				4	30	1.80	0.0600	0.5263
				5	30	1.84	0.0613	0.5158

**Table 6:** Mutual interference of *Diomus guilavoguii* feeding on young nymphs of *Paracoccus marginatus*.

Predator	Intraspecific Interference Equation	Search Constant (*Q*)	Interference Coefficient (*m*)	Predator Density	Prey Density	Prey Killed by per Predator	Predation Rate	Intensity of Scramble Competition (*I*)
Female adult	*E* = 0.2495*P*^−0.528^	0.2495	0.528	1	500	132.60	0.2652	0.0000
				2	500	78.90	0.1578	0.4050
				3	500	66.13	0.1323	0.5013
				4	500	65.10	0.1302	0.5091
				5	500	53.92	0.1078	0.5934
Male adult	*E* = 0.1799*P*^−0.446^	0.1799	0.446	1	500	92.60	0.1852	0.0000
				2	500	64.20	0.1284	0.3067
				3	500	52.07	0.1041	0.4377
				4	500	51.35	0.1027	0.4455
				5	500	44.08	0.0882	0.5240
4th instar larvae	*E* = 0.2399*P*^−0.29^	0.2399	0.29	1	200	47.00	0.2350	0.0000
				2	200	39.50	0.1975	0.1596
				3	200	36.00	0.1800	0.2340
				4	200	34.00	0.1700	0.2766
				5	200	28.00	0.1400	0.4043
3rd instar larvae	*E* = 0.2037*P*^−0.263^	0.2037	0.263	1	200	40.60	0.2030	0.0000
				2	200	33.30	0.1665	0.1798
				3	200	32.20	0.1610	0.2069
				4	200	28.20	0.1410	0.3054
				5	200	26.00	0.1300	0.3596

## Data Availability

All data is contained within the article.
